# Predicting Aging of Brain Metabolic Topography Using Variational Autoencoder

**DOI:** 10.3389/fnagi.2018.00212

**Published:** 2018-07-12

**Authors:** Hongyoon Choi, Hyejin Kang, Dong Soo Lee, The Alzheimer's Disease Neuroimaging Initiative

**Affiliations:** ^1^Department of Nuclear Medicine, Seoul National University College of Medicine, Seoul, South Korea; ^2^Department of Molecular Medicine and Biopharmaceutical Sciences, Graduate School of Convergence Science and Technology, Seoul National University, Seoul, South Korea; ^3^Korea Brain Research Institute, Daegu, South Korea

**Keywords:** brain metabolism, FDG PET, variational autoencoder, deep generative model, APOE4

## Abstract

Predicting future brain topography can give insight into neural correlates of aging and neurodegeneration. Due to variability in the aging process, it has been challenging to precisely estimate brain topographical change according to aging. Here, we predict age-related brain metabolic change by generating future brain ^18^F-Fluorodeoxyglucose PET. A cross-sectional PET dataset of cognitively normal subjects with different age was used to develop a generative model. The model generated PET images using age information and characteristic individual features. Predicted regional metabolic changes were correlated with the real changes obtained by follow-up data. This model was applied to produce a brain metabolism aging movie by generating PET at different ages. Normal population distribution of brain metabolic topography at each age was estimated as well. In addition, a generative model using APOE4 status as well as age as inputs revealed a significant effect of APOE4 status on age-related metabolic changes particularly in the calcarine, lingual cortex, hippocampus, and amygdala. It suggested APOE4 could be a factor affecting individual variability in age-related metabolic degeneration in normal elderly. This predictive model may not only be extended to understanding the cognitive aging process, but apply to the development of a preclinical biomarker for various brain disorders.

## Introduction

Understanding the normal aging change in the brain is essential to investigate neural correlates of cognitive aging and various neurodegenerative diseases including Alzheimer's disease (Jagust et al., 2009). In particular, the brain metabolism which can be measured by ^18^F-fluorodeoxyglucose (FDG) PET has been regarded as a key biomarker for neurodegenerative disorders. Identifying brain metabolic topography associated with aging could give insight into the neural basis of age-related cognitive decline and help differentiate normal aging from neurodegenerative disorders.

Although the relationship between cerebral glucose metabolism and aging has been repeatedly studied, there has been controversy about which brain regions show significant age-related metabolic decline (Duara et al., 1984; Loessner et al., 1995; Moeller et al., 1996; Petit-Taboue et al., 1998; Yanase et al., 2005). Individual genetic background and healthy status as well as underlying brain disease give rise to the individual variability in age-related metabolic change (Raz et al., 2005; Grady, 2012). Due to this variability, we have not been able to predict individual aged brain understandably. Instead of consideration of individual variability, previous studies have focused on the trend of overall aging changes using cross-sectional imaging data with statistical models such as linear regression. Even though this statistical analysis could provide overall brain metabolic changes, it was difficult to individually apply to estimate how far a given subject's brain metabolism is from the normal population at the same age. This individual evaluation of brain metabolism can be extended to the differentiation between normal and abnormal aging process. It requires normal population distribution database of all ages, however, it has been challenging to build a database of the population distribution of normal brain metabolism for each age from the limited cross-sectional data with subjects of various age distribution.

Here, we develop a model for predicting future brain metabolic topography by generating brain PET image. In this study, we utilize variational autoencoder (VAE), a type of unsupervised learning methods, which can generate images from some representations (VAE) (Kingma and Welling, 2013). We applied it to predicting FDG brain PET at different ages. Each FDG PET image combined with the subject's current age information was represented by low-dimensional features and then PET images corresponding different ages were generated. We also generated population distribution data of normal brain metabolic topography at different ages, which represented variability in individual metabolic activity at each age. As an application of our approach to discovering factors that potentially affect brain aging, we further investigated whether APOE4 status impacted on the age-related metabolic change by using a generative model that uses age and APOE4 information.

## Materials and methods

### Subjects

In this study, the data included subjects recruited in Alzheimer's Disease Neuroimaging Initiative (ADNI) with FDG PET images (http://adni.loni.usc.edu). The ADNI was launched in 2003 as a public-private partnership, led by Principal Investigator Michael W. Weiner, MD, VA Medical Center and University of California San Francisco. ADNI recruited subjects from over 50 sites across the US and Canada. The primary purpose of ADNI has been to test whether serial imaging and biological markers, and clinical and neuropsychological assessment can be combined to measure the progression of MCI and early AD. For up-to-date information, see http://www.adni-info.org. Written informed consent to cognitive testing and neuroimaging prior to participation was obtained, approved by the institutional review boards of all participating institutions. Three hundred and ninety three cognitively normal subjects without Alzheimer's dementia or mild cognitive impairment performed baseline FDG PET (Age: 73.7 ± 5.9, range 56.1–90.1). These PET data and their age information were used for developing the model. All subjects underwent the clinical and cognitive assessment at the time of acquisition. APOE genotyping was performed on DNA samples obtained from blood. For detailed information on DNA sample preparation and genotyping, see http://www.adni-info.org. For 393 subjects, 113 (28.8%) were APOE4 carriers and 280 (71.2%) were APOE4 non-carriers.

### FDG PET preparation

All the PET images were downloaded from ADNI database. FDG PET images were acquired 30 to 60 min and the images were averaged across the time frames and standardized to have same voxel size (1.5 × 1.5 × 1.5 mm). PET images were acquired in the 57 sites participating in ADNI, scanner-specific smoothing was additionally applied (Jagust et al., 2015). PET images were spatially normalized to the Montreal Neurological Institute (MNI) space using statistical parametric mapping (SPM8, www.fil.ion.ucl.ac.uk/spm). Each PET image was divided by mean FDG uptake of the cerebellum for normalization.

### Variational autoencoder for PET volumes

**Figure 1:**
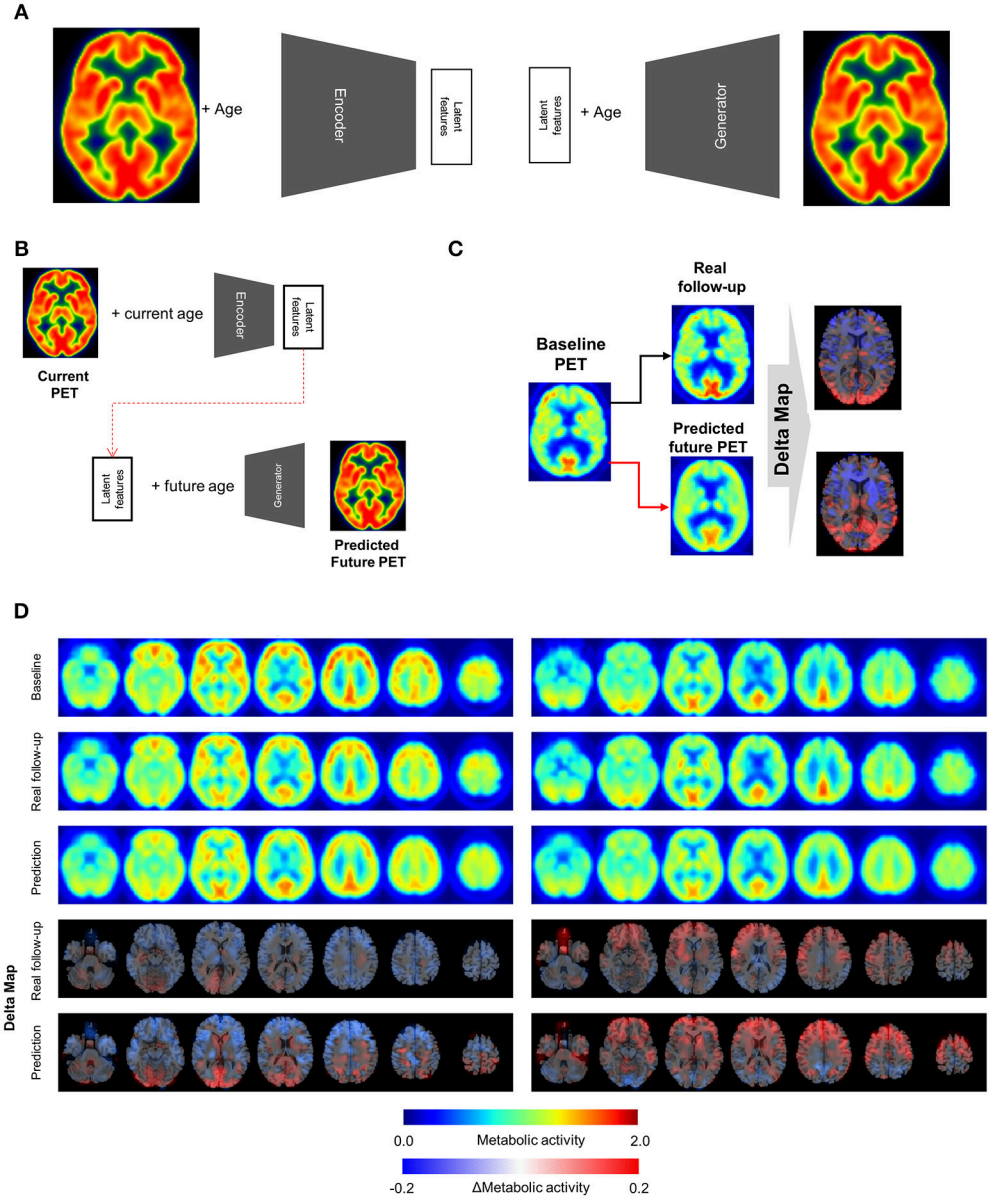
Metabolic change prediction by generating future brain PET. **(A)** VAE model which consists of encoder and generator was trained by PET images of cognitively normal subjects. The encoder represents input PET images to 10 latent features. The generator generates virtual PET image from any values of latent features and age information. **(B)** The VAE-based model could generate future brain PET individually using baseline PET image. A subject's brain PET was encoded into latent features. We hypothesized that these latent features were unchanged across age. Future brain PET was generated by entering future age and the latent features. **(C)** Predicted individually generated PET was compared with real follow-up data. For comparison, delta maps obtained by subtracting baseline from prediction or follow-up images were generated. **(D)** Representative cases follow-up PET and individually predicted PET. According to the follow-up data, there was comparable individual variability in metabolic change. A subject showed globally decreased metabolism (left) while another subject showed increased metabolism in the frontotemporal cortex (right). Predicted future PET could also reflect the individual variability.

We utilized VAE model to generate virtual PET data according to age information. VAE-based PET image generation is summarized in Figure 1A. VAE is a type of unsupervised learning methods which could represent the high-dimensional data to low-dimensional features. The major strength of the VAE is to generate virtual data from latent features. VAE consisted of two components, encoder and generator. The encoder reduces the dimension of data by compressing them to latent features and the generator produces the data from any values of latent features. The generator of VAE is a probabilistic generator which assumes that the data were generated from some conditional distribution and an unobserved variable *z* in latent space. Thus, the probabilistic generator can be defined by *p*_θ_(*x*|*z*). θ represents the parameters of generator. The posterior distribution *p*_θ_(*z*|*x*) can be obtained by prior distribution *p(z)*, *p*_θ_(*z*|*x*)~ *p*(*z*)*p*_θ_(*x*|*z*). Variational Bayes learns both parameters, *p*_θ_(*x*|*z*) and an approximation *q*_ϕ_(*z*|*x*) to the intractable true posterior *p*_θ_(*z*|*x*). This is achieved by the loss function,

$$\begin{array}{l} L\left(\phi ,\theta \right)=\;-E_{z\sim q_{\phi }\left(z|x\right)}\left(\log p_{\theta }\left(x|z\right)\right)+\;KL\left(q_{\phi }\left(z|x\right)∥p_{\theta }\left(z\right)\right) \end{array}$$

where KL is Kullback-Leibler divergence between the learnt latent distribution and the prior distribution *p*_θ_(*z*), acting as a regularization term (Kingma and Welling, 2013). The first term represents reconstruction loss of autoencoder.

In this study, we applied VAE with age information to generate PET image, so used VAE conditioning on another description of the data, *y* (i.e., age information). This model is aimed to generate data from the conditional distribution as well as latent features *z*. Thus, the probabilistic generator and the encoder can be defined by *p*_θ_(*x*|*y, z*) and *q*_ϕ_(*z*|*x, y*), respectively. The loss function is changed to,

$$\begin{array}{l} L\left(\phi ,\theta \right)=\;-E_{z\sim q_{\phi }\left(z|x,y\right)}(\log p_{\theta }(x|y,z))+\;KL(q_{\phi }\left(z|x,y\right)\|\\ \;p_{\theta }\left(z\right)) \end{array}$$

To train VAE, data *X* and age information *y* were encoded into parameters in a latent features *Z*, and decoder network reconstructs data from the latent features and *y* assuming latent features have normal distributions around encoded feature *z*. In practice, generator input was resampled by the encoded latent features *z* assuming normal distribution:*z*_*resampled*_ = *z*_*encoder*_ + *z*_*sd*_ × ε, where ε represents a random variable (Kingma and Welling, 2013).

### Network architecture and training

To encode 3-dimensional PET volume, we used multiple 3D convolutional layers for encoding. Specific parameters for network architecture are summarized in Supplementary Figure 1. After the multiple convolutional and pooling layers, 3D feature volumes are changed to 1-dimensional features. These features are merged by age information of each subject and additionally connected to hidden layers and, finally, connected to 10 latent features. Accordingly, initial PET volume with 79 × 95 × 68 matrix is compressed into 10-dimensional features. Conversely, the generator consists of convolutional and upsampling layers. Upsampling simply repeats each dimension of the data. Input variables of the generator include 10 latent features and age information. The generator decodes these inputs to PET volume.

This conditional VAE model was trained by gradient descent algorithm (Adadelta) (Zeiler, 2012) and took 50 epochs for the training. The VAE was implemented using a deep learning library, Keras (ver. 1.2.2) with Theano (ver. 0.9.0) backend (Bastien et al., 2012). Ten percentage of all PET data were used for the validation set to determine epoch number and hyperparameters for the neural network architecture.

### Estimation of metabolic activity in brain regions

The regional metabolic activity of brain regions was obtained using predefined volume-of-interests (VOI), automated anatomical labeling (AAL) template. As all PET images were spatially normalized to MNI template, mean metabolic activity value of each brain region was simply obtained by masking specific brain region.

### Prediction of future PET and comparison with follow-up PET

Four-year follow-up FDG brain PET scans were obtained in 26 cognitively normal subjects who underwent baseline PET scans. Five-year follow-up FDG brain PET scans were acquired in 11 cognitively normal subjects. Longitudinal change in brain metabolism was evaluated in these subjects. Using baseline PET images of the subjects and age, we generated future PET images. To generate individual future PET image, firstly, baseline PET image was represented into latent features using the encoder. We hypothesized that these latent features were unchanged regardless of subject's age. Ten latent features of a subject and future age (i.e., baseline age + 4 or 5) were used for the generator. We compared real follow-up PET and predicted PET by using delta maps. To measure similarity between predicted and real metabolic changes, voxelwise correlation coefficient was calculated. Similarity measurements were individually obtained. We statistically tested whether other variables including baseline age, gender, APOE4 status, Mini-Mental State Examination (MMSE) and follow-up diagnosis affected the prediction of metabolic changes. The similarity measurements, correlation coefficients, of the group according to the APOE4 status, gender and follow-up diagnosis were statistically compared using independent *t*-test. They were correlated with continuous variables (age and MMSE) using Pearson correlation. We also additionally evaluated the overall accuracy of predicted image using mean absolute percentage error (MAPE). MAPE between predicted and real follow-up PET image was calculated for each subject.

In addition, overall predicted and real regional changes were calculated by AAL map. The overall regional metabolic change was calculated by mean value across all subjects. The correlation between regional metabolic changes of predicted and real follow-up PET across brain regions was tested by Pearson correlation. For visualizing the similarity between predicted and real metabolic changes, Bland-Altman plots were drawn. Ninety percentage confidence interval for error of predicted regional metabolic change was calculated.

### Generation of age-related metabolic change movie

The overall age-related metabolic change pattern was evaluated by the generator model. Firstly, PET data of all subjects were represented by 10 latent features using the encoder. The mean feature values were entered into the generator with different age information between the age of 50 and 100. Thus, we could obtain representative PET image of each age. To visualize age-related metabolic change, we generate subtraction map. Generated PET images with different age were subtracted by a representative brain PET generated by age of 50. These subtraction maps were also visualized by an animation.

### Population distribution of regional metabolic activity at each age

We estimated population distribution of regional metabolic activity by resampling generated PET images. Ten latent features were randomly resampled assuming each latent feature has normal distribution. Mean and standard deviation of each latent feature were determined by the feature values of all subjects. One Thousand resampled brain PET images were generated and regional metabolic activity was obtained. Population distribution of metabolic activity of each region was drawn by histograms and age-related changes with confidence intervals were drawn.

### Metabolic topography according to latent features

To assess the relationship between latent features and brain metabolic patterns, brain PET images were generated by changing values of the latent features. Mean values of latent features were used for generating PET except for two features for estimating effects on brain metabolism. These two features were changed from −2.0 to 2.0 and generated virtual PET images for plotting.

### Variability in age-related metabolic change according to the APOE4 status

To evaluate age-related metabolic change patterns according to the APOE4 status, another VAE model was trained. Conditional VAE with age and APOE4 status information was used, so, conditional variable, *y*, includes age and APOE4 status as different dimensions. The training process and network architectures were same with conditional VAE with age only.

The overall age-related metabolic change patterns according to APOE4 status was evaluated as population distribution estimation. Randomly resampled latent features and different age values were entered into the generator with each APOE4 status respectively. PET images of each age and APOE4 status were generated and regional metabolic activity was obtained by predefined regions. Population distribution of regional metabolic activity was estimated for APOE4 carriers and non-carriers. To find statistically different regions, we calculated the difference between regional metabolic activity generated by APOE4 carriers and non-carriers using randomly resampled latent feature values. To define statistical significance, *p*-values were computed by the distribution of the difference. The null hypothesis was that the regional metabolic difference between APOE4 carriers and non-carriers is 0. Thus, the statistical significance could be directly calculated by the proportion of the generated samples where the difference was lower or higher than 0. Brain regions with different metabolic activity were found at each age. The difference with uncorrected *p*-value < 0.05 was regarded as significant brain regions which show different metabolism according to APOE4 status.

## Results

### Prediction of future brain metabolic change

The VAE-based model was designed to represent FDG PET images and corresponding subjects' age to latent features (Figure 1A). The posterior part of this model, the generator component, could produce PET images from any values of the latent features and age information.

To generate future brain PET images, we firstly obtained latent features of a subject's baseline PET image using the encoder. We assumed that these were not changed according to aging as characteristic individual features. The features of a subject were entered into the generator with any age, which could generate the subject's virtual brain PET at different ages (Figure 1B). The model was tested by cognitively healthy subjects who underwent both baseline and follow-up PET. The predicted metabolic change was compared with corresponding real metabolic change computed by follow-up PET data. Each predicted future brain PET and real follow-up PET was subtracted from corresponding baseline PET for the comparison (Figure 1C). As a result, delta maps, the future brain PET subtracted by the baseline, obtained from real follow-up PET showed individual variability. Corresponding predicted future brain PET also showed those variable patterns (Figure 1D). A subject showed prominently decreased metabolism in the cerebral cortices, while another showed relatively increased metabolism in the frontal cortex (Figure 1D). The delta map obtained by real follow-up was positively correlated with that obtained by prediction (Supplementary Figure 2).

**Figure 2:**
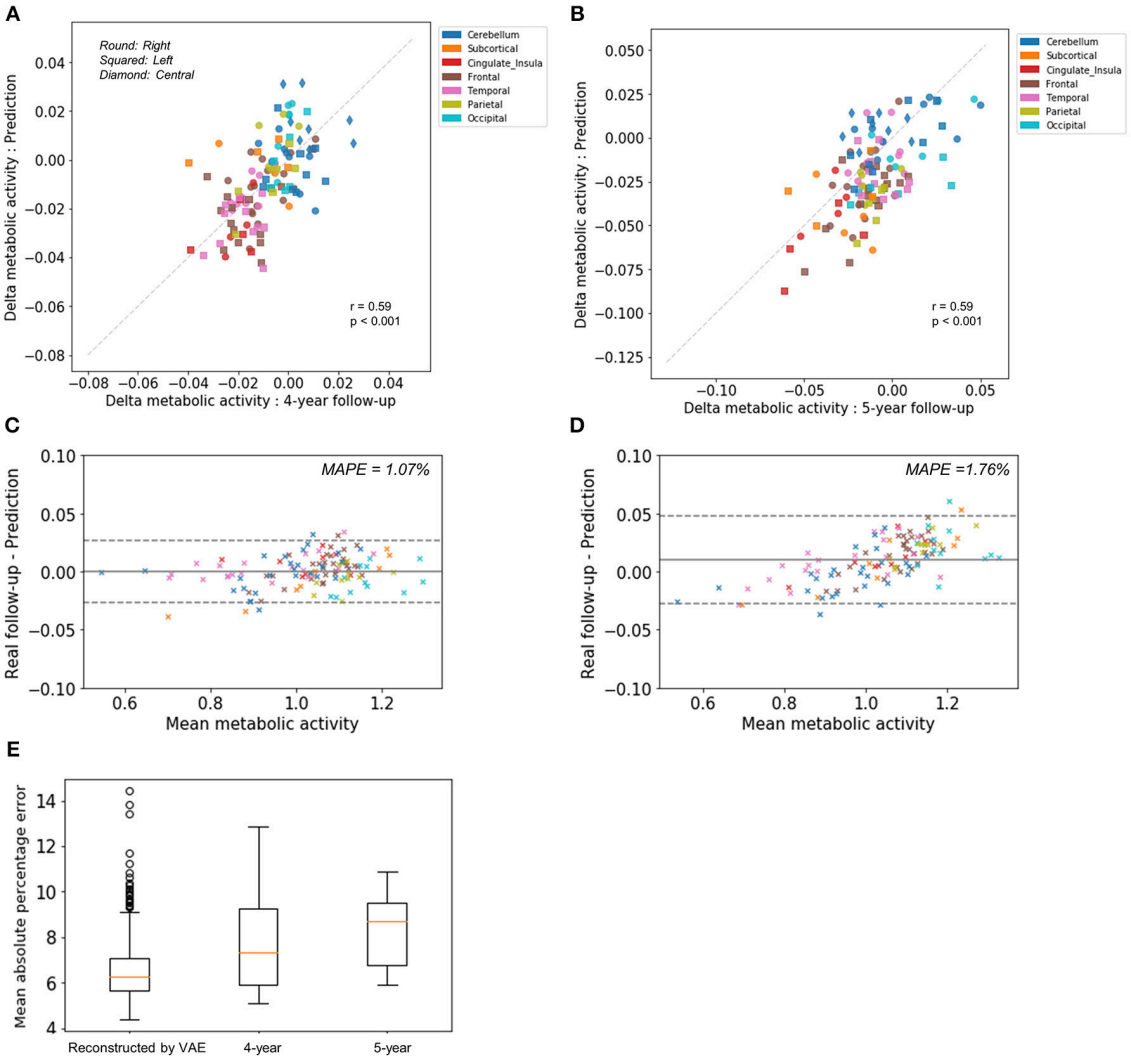
Comparison of predicted metabolic change with real follow-up data. Regional metabolic change from baseline was averaged across subjects for predicted and follow-up data. Averaged predicted and real changes across the brain regions were significantly correlated for 4-year follow-up images (*r* = 0.59, *p* < 0.001) **(A)** and 5-year follow-up images (*r* = 0.59, *p* < 0.001) **(B)**. Bland-Altman plots were drawn for the comparison of predicted and real regional metabolic activity for 4-year **(C)** and 5-year PET images **(D)**. The 95% confidence interval of the error of predicted regional metabolism was −0.027 to 0.027 for 4-year follow-up and −0.027 to 0.048 for 5-year follow-up. Mean absolute percentage error (MAPE) was 1.07% for 4-year follow-up and 1.76% for 5-year follow-up. **(E)** As a global measurement of accuracy for predicting future brain PET, MAPE was 7.8 ± 2.1% for 4-year follow-up and 8.3 ±1.5% for 5-year follow-up. MAPE calculated by baseline PET and output of VAE with baseline age was 6.6 ± 1.4%.

To compare predicted future brain PET and real follow-up PET quantitatively, mean metabolic changes of 116 predefined brain regions across all subjects were calculated. Averaged predicted changes in regional metabolism was significantly correlated with the real changes obtained by real follow-up data (*r* = 0.59, *p* < 0.001 and *r* = 0.59, *p* < 0.001 for 4-year and 5-year follow-up, respectively; Figures 2A,B). Bland-Altman plots showed the difference between predicted and real regional metabolic activities (Figures 2C,D). The 95% confidence interval of the prediction error of regional metabolic activity was −0.027 to 0.027 for 4-year follow-up and −0.027 to 0.048 for 5-year follow-up. In addition, individually predicted and real metabolic changes were compared. To show how individual prediction of metabolic change was similar to the real change, voxelwise correlations of individual delta maps obtained by follow-up and prediction were calculated. We could find a trend of high correlation between the two delta maps of the same subject, even though the prediction of metabolic change was failed in some subjects (Supplementary Figure 3). The similarity between predicted and real metabolic change was not significantly affected by subjects' age, gender, follow-up diagnosis, APOE4, and baseline MMSE. As a global measurement of overall accuracy for predicting future brain PET, we obtained MAPE by comparing predicted PET with real follow-up PET. MAPE was 7.8 ± 2.1 and 8.3 ±1.5% for 4-year and 5-year follow-up, respectively. Notably, MAPE calculated by baseline PET and reconstructed PET using VAE was 6.6 ± 1.4% (Figure 2E).

### Generating overall brain metabolism aging movie

**Figure 3:**
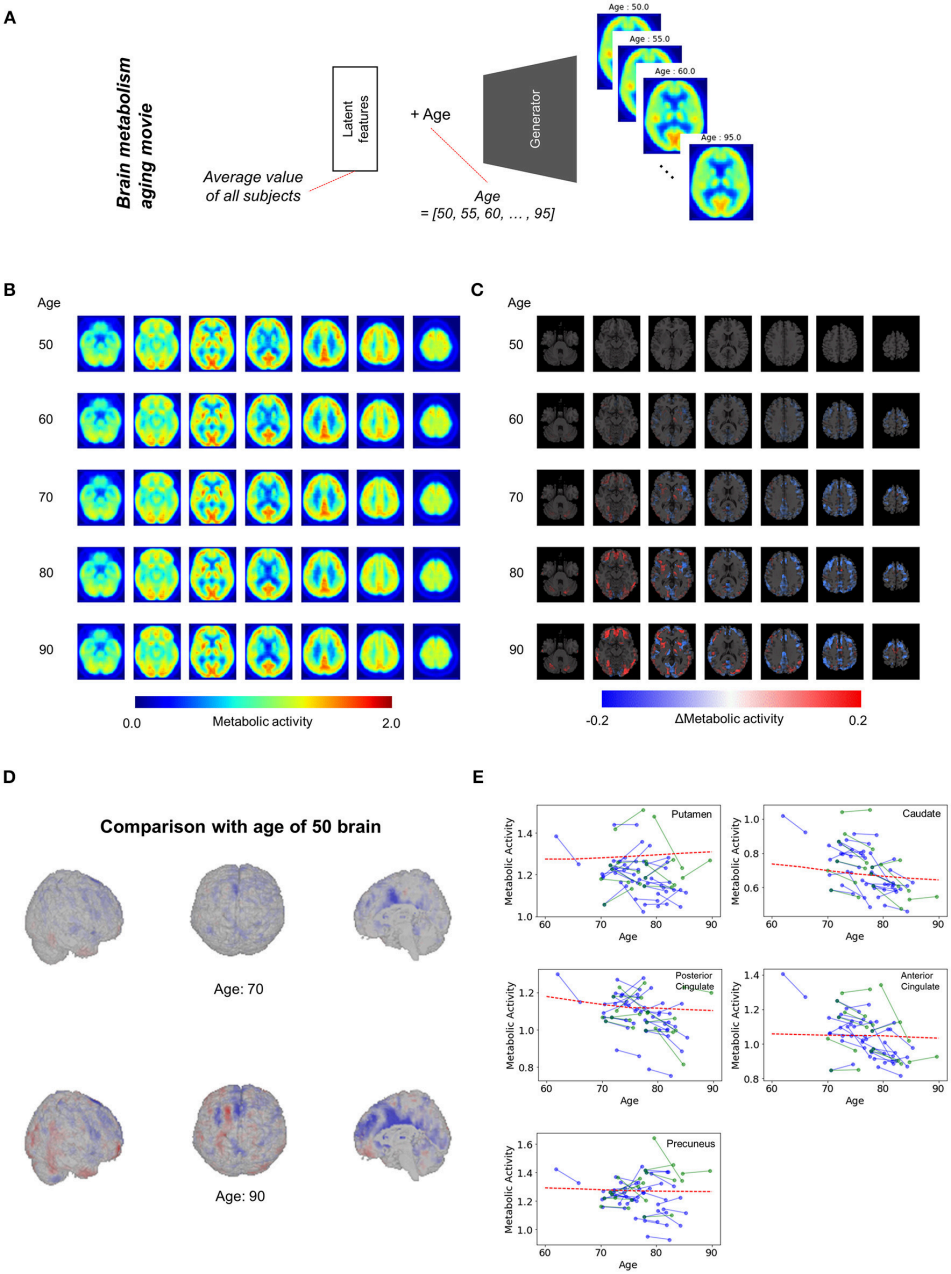
Overall brain metabolism aging movie by generating representative PET of each age. **(A)** Using VAE-based model, representative FDG PET images of different age were generated to identify overall age-related metabolic pattern. Mean latent feature values across all trained subjects were entered into the generator for representative PET images. **(B)** Using mean latent features, representative PET images were generated according to aging. **(C)** Compared with the representative PET of age of 50, subtraction images were generated. **(D)** Surface visualization of the subtraction map revealed that age-related decline was mainly found in the cingulate cortex. **(E)** Age-related metabolic change in specific brain regions was plotted. Solid lines represent real metabolic change data for 4-year follow-up (blue) and 5-year follow-up (green). Red dotted lines represent regional metabolic changes estimated by virtually generated PET images.

We applied our model to the assessment of overall regional metabolic changes. To investigate overall patterns of age-related brain metabolism, representative brain images were generated by using different age and mean value of each latent feature across all subjects (Figure 3A). The representative FDG brain PET generated from the age of 50 to 90 is presented in Figure 3B. To visualize the age-related change definitely, the generated FDG PET with different age was subtracted by the generated PET of the age of 50 (Figures 3C,D, Supplementary Figure 4). As we generated brain metabolic topography at all ages, overall age-related patterns were also visualized by movies (Supplementary Movies 1, 2).

Figure 3D showed that age-related metabolism decline was mainly found in the cingulate cortex. Using predefined brain regions of interests, the metabolic activity of each brain region was extracted according to aging (Figure 3E). Red dotted lines represent estimated metabolic decline using the generated PET obtained by entering mean latent features. Solid lines represent real metabolic decline obtained by 4-year (Blue) and 5-year (Green) follow-up data (Figure 3E). The curves estimated by the VAE model explained that overall metabolic decline with aging was non-linear. Approximately before 75, the age-related metabolic decline was steep in the posterior cingulate and caudate and then the decline became slower after 75.

### Distribution of regional metabolic activity at each age

**Figure 4:**
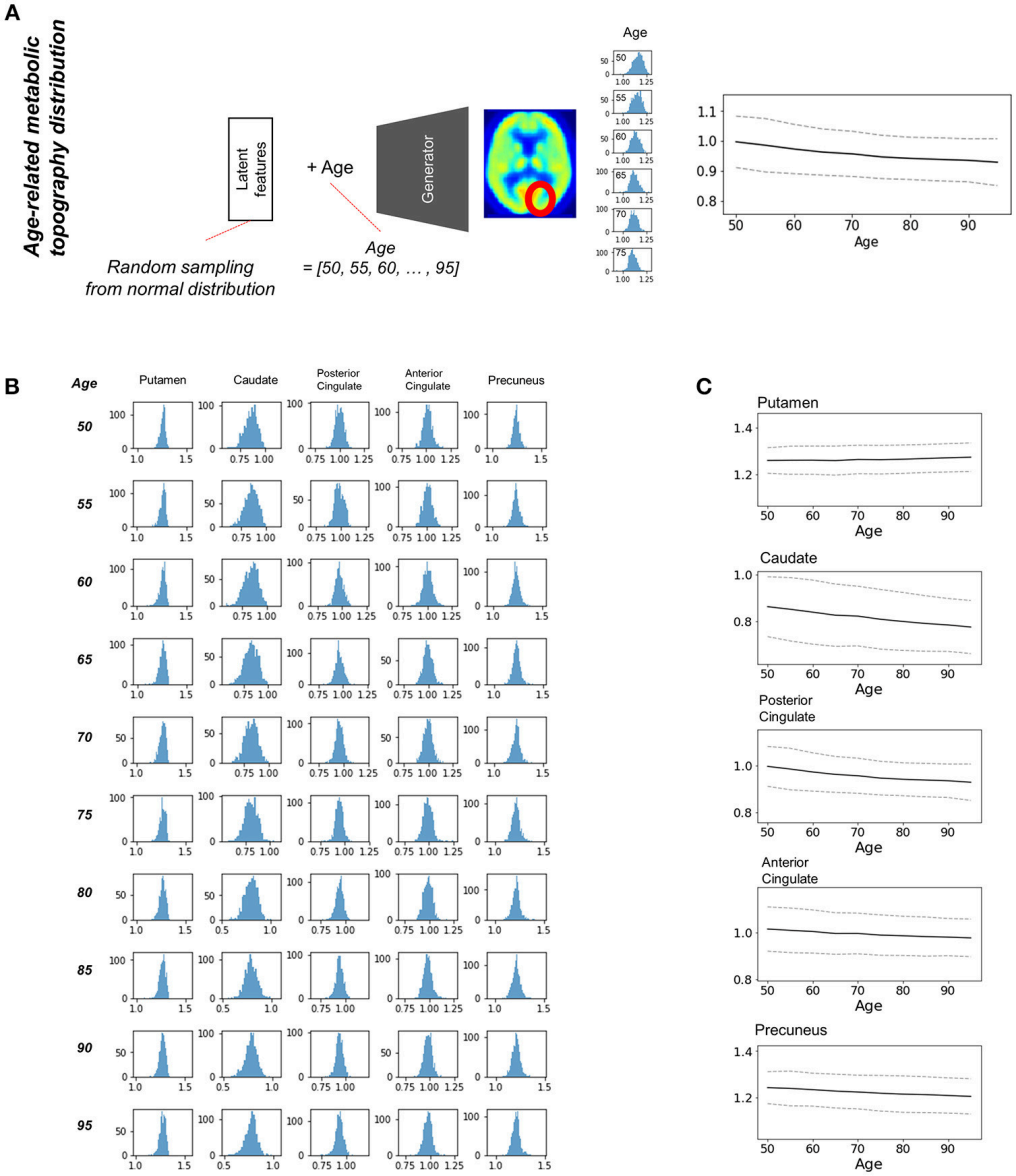
Estimating population distribution of brain metabolism at each age**. (A)** Population distribution of brain metabolic topography was estimated by resampling latent features. Generated brain PET was repeatedly generated by random latent feature values sampled from the normal distribution. Distribution of regional metabolism was estimated for all ages. **(B)** Histograms of distribution of metabolic activity were drawn for putamen, caudate, posterior cingulate, anterior cingulate, and precuneus at different ages. **(C)** Confidence intervals of metabolic changes could be estimated by the distribution. Dotted lines represent 95% confidence interval of regional metabolic activity.

Most brain imaging data including our subjects consist of imaging with various ages. Thus, it has been challenging to obtain population distribution of normal brain at each age. Randomly resampled latent features could generate population distribution of regional brain metabolic activity for all ages (Figure 4A). Generated brain PET data from resampled latent features provide the variety of regional metabolic activity. Histograms of each brain region at different ages were drawn (Figure 4B). As aforementioned representative brain metabolic changes, histograms of posterior cingulate and caudate showed a trend of left shifting according to aging. Distribution of overall aging patterns of regional metabolism was also exhibited (Figure 4C, Supplementary Figure 5). Dotted lines represent 95% confidence intervals of regional metabolic activity.

**Figure 5:**
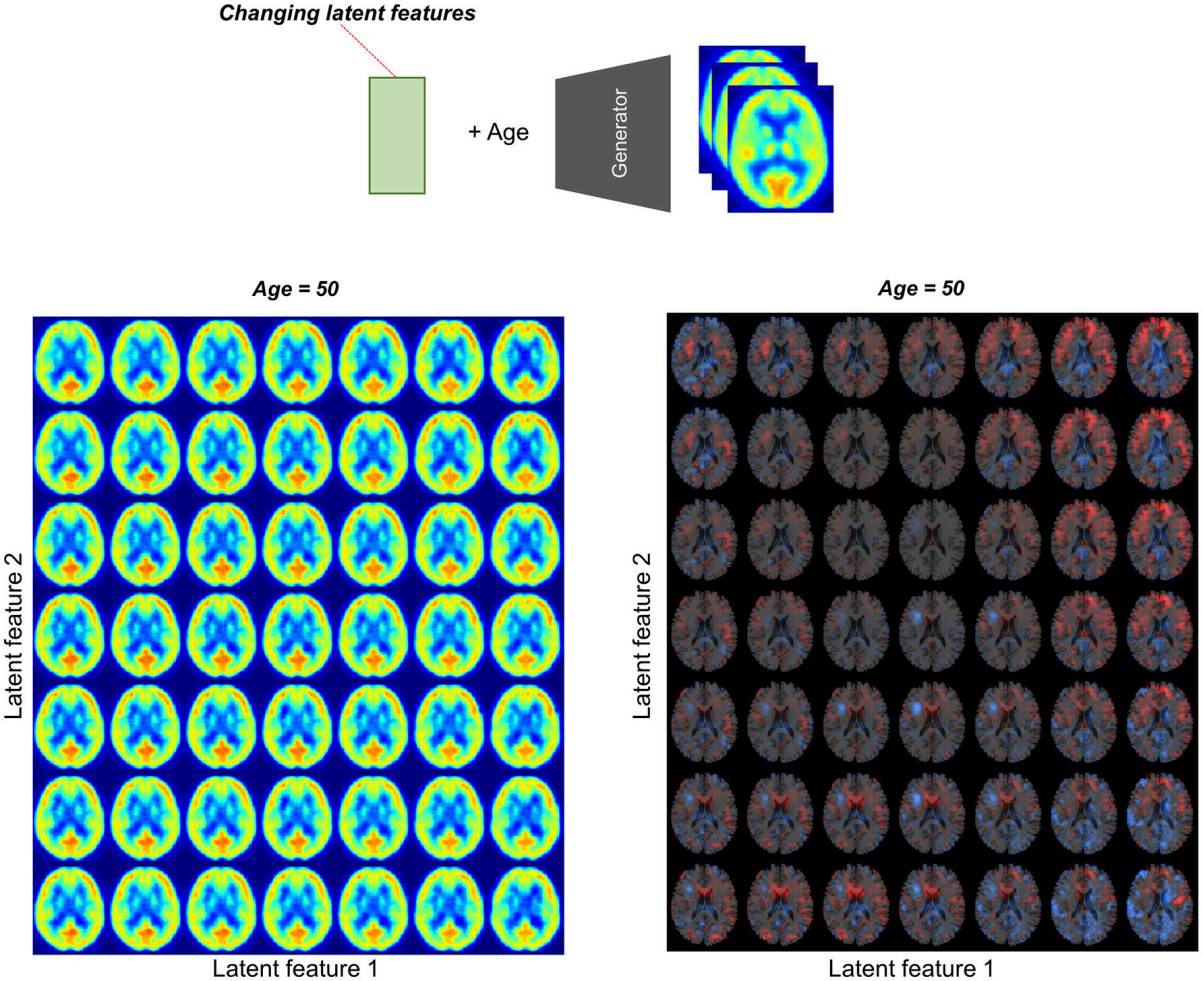
Brain metabolic topography according to latent features. As the encoder of VAE compressed PET image into 10 latent features, variability in brain metabolism is determined by these 10 features. To assess metabolic patterns determined by latent features, brain PET images were generated according to different latent feature values. An example of the two latent features, increased first latent feature (x-axis) was associated with decreased metabolism in posterior temporal and occipital cortices. Increased second latent feature (y-axis) was associated with increased metabolism in the frontotemporal cortices at age of 50.

We found individual variability in regional brain metabolism at different ages. The individual variability was determined by the distribution of latent features. To show how each latent feature affects brain metabolism, PET images were generated by changing latent features. Brain metabolic patterns were changed according to latent features as shown in Figure 5. As an example, increased feature 1 was associated with decreased brain metabolism in the posterior temporal and occipital cortices and increased feature 2 was associated with increased frontal metabolism.

### APOE4 status and age-related metabolic change

**Figure 6:**
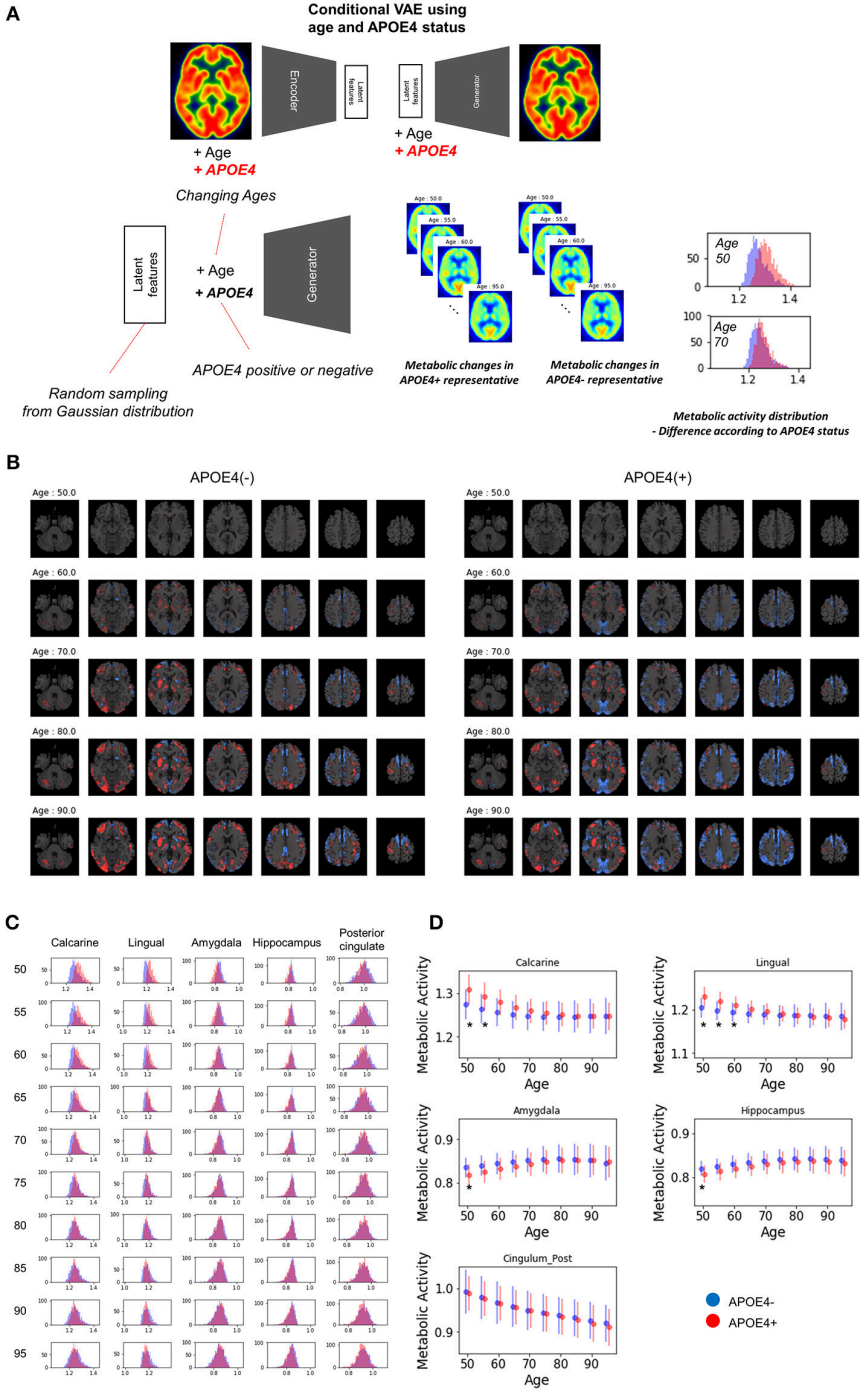
APOE4 status and age-related brain metabolic change**. (A)** We investigated whether APOE4 status affect age-related metabolic change patterns. A conditional generative model was developed using APOE4 status as well as age. PET images according to different ages were generated for APOE4 carrier and non-carrier, respectively. Resampled features provide distribution difference of brain metabolic topography between APOE4 carriers and non-carriers. **(B)** Delta maps were generated by subtracting 50-year-old generated images. Metabolic decline was relatively faster in occipital regions of APOE4 carrier. **(C)** Histograms of regional metabolic activity were drawn for APOE4 carriers and non-carriers. Before 60, metabolism of calcarine, lingual cortex, hippocampus, and amygdala was different according to APOE4 status. **(D)** Age-related regional metabolic activity changes were plotted. Red dots represented APOE4 carriers and blue dots represented APOE4 non-carriers. Bars represented standard deviations calculated by the distribution. Non-parametric testing revealed the statistical significance. Asterisks represent uncorrected *p* < 0.05.

Because clinical variables affect age-related metabolic change and its variability, we further investigated whether APOE4 status impacts on metabolic changing patterns. Another VAE model was trained using two conditions, age and APOE4 status (Figure 6A). This model can generate virtual brain PET images according to the age and APOE4 status. Thus, age-related metabolic change according to APOE4 can be estimated by inputting APOE4 positive and negative states, respectively (Figure 6A). We identified that APOE4 could affect the variability of age-related metabolic change. The FDG PET images generated by average latent features and APOE4 positive and negative status at different ages were subtracted by the generated PET of the age of 50 (Figure 6B). We found that metabolic decline in occipital lobe was faster in APOE4 carriers. Distribution of regional metabolism according to APOE4 status was estimated (Figure 6C). Using distribution of the metabolic difference between brain metabolism generated by APOE4 status, the significance of the difference in regional metabolism was estimated (Supplementary Figure 6). The regional metabolic activity of the calcarine and lingual cortex was significantly higher in APOE4 carrier than APOE4 non-carrier before 60, while that of the hippocampus and amygdala was significantly lower in APOE4 carrier at 50 (Figure 6D). The regional metabolic activity of posterior cingulate, precuneus and caudate, where the rapid age-related metabolic decline was found, did not show a significant difference in accordance with APOE4 status. Metabolic change in APOE4 carriers and non-carriers of all brain regions was represented with 95% confidence intervals (Supplementary Figure 7).

## Discussion

In this study, we predicted aging of brain metabolic topography by using a generative model. Brain metabolic changes are highly variable as aging process and cognitive changes are affected by several individual factors. Our model aimed at generating PET images according to the age trained by cross-sectional PET image data combined with different ages. The model could provide predicted future metabolic decline and was validated by real follow-up data. Our results estimate population distribution of normal brain metabolism at each age. This approach was extended to investigate the effect of APOE4 status on the variability of regional brain metabolism at different ages.

Our generative model could find population distribution of brain metabolic topography for each age as well as predict age-related metabolic change. Cognitive aging and the age-related functional decrease are accompanied by increased individual variability (Ylikoski et al., 1999). This individual variability is affected by several factors including life experience, genetic backgrounds, and susceptibility to neuropathology (Shammi et al., 1998). Furthermore, cognitive variability in individuals across time tends to occur mainly after the age of 60 (Wilson et al., 2002). Increased individual variability in aging has been supported by several functional neuroimaging studies (Glisky et al., 2001; D'esposito et al., 2003; Burzynska et al., 2015). Nonetheless, age-related brain metabolism change has been briefly estimated by observing an overall correlation between age and metabolism (Duara et al., 1984; Loessner et al., 1995; Moeller et al., 1996; Petit-Taboue et al., 1998; Yanase et al., 2005). This previous approach could not consider individual variability in age-related metabolism (Ylikoski et al., 1999; Knopman et al., 2014). Furthermore, it has been difficult to estimate age-dependent normal population distribution of brain image data as the data consist of subjects with different ages. A conventional linear regression model based on overall metabolic changes estimated by all baseline scans only estimated same decline patterns for all subjects by calculating a voxelwise linear regression based on the population.

According to our model, the individual variability of brain metabolism was represented by the latent features. The latent features determine age-related metabolism patterns because the generator used only the latent features and age as inputs. We could indirectly know whether the VAE model uses age information for generating PET images. Firstly, the generator of VAE model uses age information in that different metabolism distributions are shown by different age inputs and same latent feature values. Furthermore, we compared our VAE model with another VAE model without age information, which encodes latent features regardless of age and generates PET from latent features without age inputs. As a result, the VAE model without age information extracts more age-dependent latent features than the VAE model with age information (Supplementary Figure 8). It suggests that the VAE model without age information is prone to extract age-dependent image features with unsupervised manner because age-related changes largely contribute to the variability in brain metabolism. On the other hand, our model extracts individual characteristic image features relatively independent of age by using age information for the encoder. Each latent feature represented specific metabolic topography patterns which could be indirectly identified by generating images according to different feature values as shown in Figure 5. In this regard, random resampling of the latent features generated variable brain metabolic topography, which could be used for estimating population distribution. Our result, population distribution of brain metabolism at each age can be applied to quantitatively define regional abnormality in individuals. Using this distribution, we can define how far a given individual brain PET is from the normal population. Thus, this distribution may help to develop quantitative biomarker which represents abnormal aging process of individual brain metabolism.

Our model could predict regional patterns of individual future brain metabolic change, while future prediction of metabolic change was incorrect in quite a few cases. As shown in Supplementary Figure 3, the predicted delta maps were not correlated with real delta maps in individuals at right-lower portions of the matrix. Individual age-related changes measured by PET could be the sum of biologic metabolic change and statistical random errors in FDG PET. The statistical variability in brain metabolism could affect prediction accuracy of metabolic changes. Nonetheless, overall regional metabolic changes obtained by the prediction were highly correlated with those of real follow-up data as shown in Figure 2. That was because VAE eventually extracted age-associated metabolic topography patterns from overall variation of brain metabolism in the training samples. In other words, because of the high variability in age-related brain metabolic changes, VAE-based model generated future brain PET image by approximating global age-related patterns of training samples. It is closely related to the limitation of VAE which tends to generate averaged and blurry images and lack of variety in generated images (Dosovitskiy and Brox, 2016). Notably, though predicted overall regional metabolic changes in 5-year follow-up were significantly correlated with real follow-up data, they tend to underestimation in the regions with high metabolism and overestimation in those with low metabolism (Figure 2D). MAPE of 5-year follow-up was higher than 4-year follow-up as well as reconstructed images, which suggested the prediction accuracy could be affected by follow-up intervals. It could be due to long follow-up interval which could cause more non-aging factors affecting brain metabolism. Not only aging but several cognitive, healthy, and nutritional factors affect brain metabolic patterns (Belanger et al., 2011; Cunnane et al., 2011). Because of the multiple factors affecting brain metabolism, accurate individual prediction, particularly for long-term prediction, is substantially difficult. In this study, we simply assumed that other factors of future brain PET except age are unchanged. As multiple factors could determine metabolic topography, the generative model with multiple conditions such as cognitive score may improve future PET prediction.

Combination of another generative model such as generative adversarial model may improve the prediction accuracy (Goodfellow et al., 2014). Briefly, the generative adversarial model is another generative model using two networks, generator and discriminator. The generator is trained to synthesize images from latent features which cannot be discriminated from the training data, while discriminator is trained to discriminate real images from generated images. This type of model also can be combined with conditions such as aging information. The generative adversarial model can generate more realistic images compared with VAE, however, according to our experiments, 3-dimensional PET images were hard to generate using it. A further modification will be required to train the model and to generate more accurate future brain images. In addition, parameters including the number of latent features, model architectures and optimization methods could be modified to obtain better results. Although we tested several models, methods to develop optimized neural network architectures will be required as a future work.

Population distribution of metabolic topography revealed that APOE4 carriers showed higher metabolism in the calcarine and lingual cortex, while lower metabolism in the hippocampus and amygdala before 55. The difference in these regions was not found after 60, which suggested that age-related metabolic changes of these regions were greater in APOE4 carriers than non-carriers. The relationship between APOE4 and brain metabolism in normal elderly has been investigated in previous studies as well (Oh et al., 2014). The regions which showed difference metabolism in accordance with APOE4 status were partly different as the previous study showed that metabolic decline was faster in composite region-of-interests including posterior cingulate, precuneus, and lateral parietal cortices (Oh et al., 2014). Besides, another study using functional MRI showed APOE4 status affected the differentiation of functional networks including hippocampal and visual networks though they used different modality (Trachtenberg et al., 2012). Structural MRI study showed that APOE4 carriers tended to have thicker cortex in temporooccipital areas and a steeper age-related decline in cortical thickness (Espeseth et al., 2008). Although the regions related to APOE4 were partly different according to the studies, our result supports APOE4 carriers could affect functional brain aging patterns. Additionally, by estimating population distribution, we could identify regional metabolic difference at all ages. Our approach can be extended to the investigation of the association between other clinical variables and age-related changes. It can eventually help find the factors that determine the individual variability in aging.

To our knowledge, this is the first report that applies a generative model to estimate aging of high dimensional medical data. As an extended application of our approach, PET data according to interpretable features, such as sex and cognitive scores, can be generated by using conditional VAE which aimed at synthesizing virtual data from the conditional distribution (Kingma et al., 2014; Sohn et al., 2015). This conditional generative model can be used for various problems in neuroimaging analyses. For example, the model may be used for predicting several task-specific functional brain images from a single image data. Virtual task-related brain images can be predicted by inputting tasks as conditional inputs of VAE model. Furthermore, this approach would improve conventional statistical voxelwise analyses of neuroimaging data. An important limitation of the voxelwise analysis is the presence of multiple covariates (Friston et al., 1994; Petersson et al., 1999). So far, covariates such as subject's age and brain volume have been handled as nuisance variables using general linear model. Instead, virtual neuroimaging data in same conditions can be generated by this approach. For instance, we can compare brain images of different groups by generating virtual data with controlled covariates such as same age and brain volume.

As a deep generative model may be able to precisely predict high dimensional data, the future application will be extended to various medical implications. Recently, generative models have been used in various biomedical fields as well as neuroimaging data. A generative model was applied to generating novel molecular fingerprints as an artificial intelligence drug discovery framework (Kadurin et al., 2017). As a recently developed application to medical image processing, a generative model was used for automatic lesion segmentation (Alex et al., 2017).

In our study, we predict aging of metabolic topography by generating PET images. In spite of individual variability in age-related change, future regional metabolic changes were precisely predicted. Population distribution of normal brain metabolism at different ages was estimated. It revealed that regional metabolic decline was different according to the APOE4 status. This brain metabolic change prediction method can provide a plausible explanation of individual variability in cognitive aging. Furthermore, we expect that this approach will be extended to the development of a preclinical biomarker for several neurodegenerative disorders as well as defining abnormal brain aging.

## Author contributions

HC and DL designed the study. HC generated the model. HC and HK performed statistical analysis. All authors interpreted the data, wrote, and approved the manuscript.

### Conflict of interest statement

The authors declare that the research was conducted in the absence of any commercial or financial relationships that could be construed as a potential conflict of interest.
