# Supplementary material for: Predicting Aging of Brain Metabolic Topography Using Variational Autoencoder
Source: Front Aging Neurosci. 2018 Jul 12;10:212. doi: 10.3389/fnagi.2018.00212 (PMC6052253; doi:10.3389/fnagi.2018.00212)
Supplement: Supplementary file 3 [file Image_1.PDF]

Supplementary Material

Predicting aging of brain metabolic topography using variational autoencoder

Hongyoon Cho*, Hyejin Kang, Dong Soo Lee*, for the Alzheimer's Disease Neuroimaging Initiative

*** Correspondence:** Hongyoon Choi, chy1000@snu.ac.kr; Dong Soo Lee, dsl@snu.ac.kr

1 Supplementary Figures

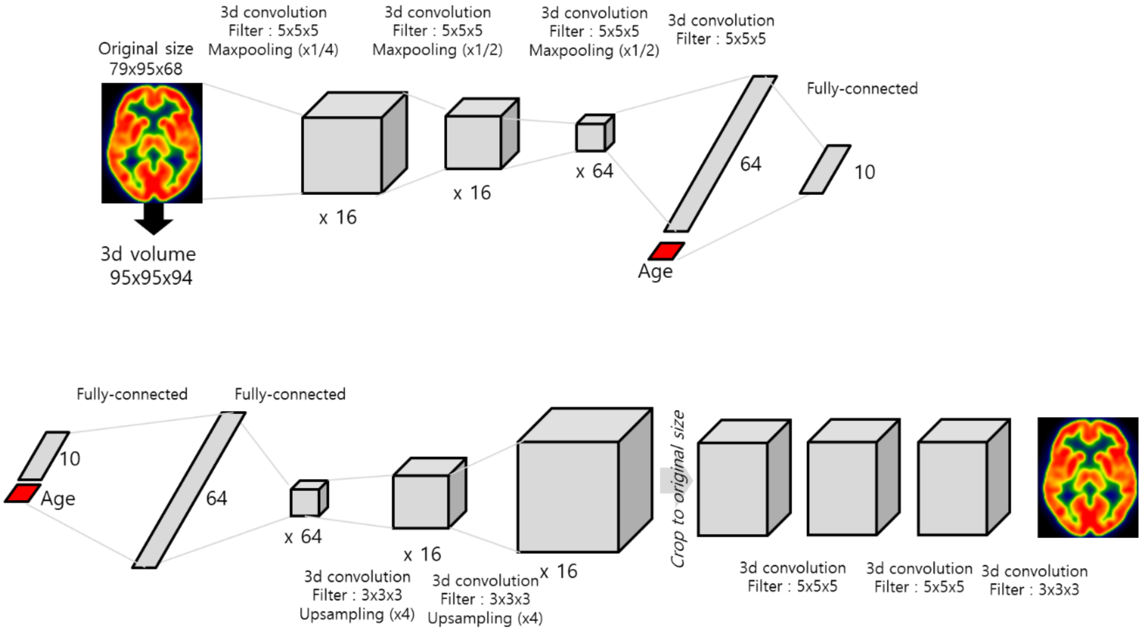

Supplementary Figure 1. Network architecture of variational autoencoder model. 3d convolutional layers followed by max-pooling layers were extracted 3-dimensional features of the PET images. After the convolutional layers, features were concatenated to another input, age information. A fully connected layer connected them to 10 latent features. The input of the generator was 10 latent features and age information. Multiple convolutional layers with upsampling layers decoded the features to 3d PET volumes.

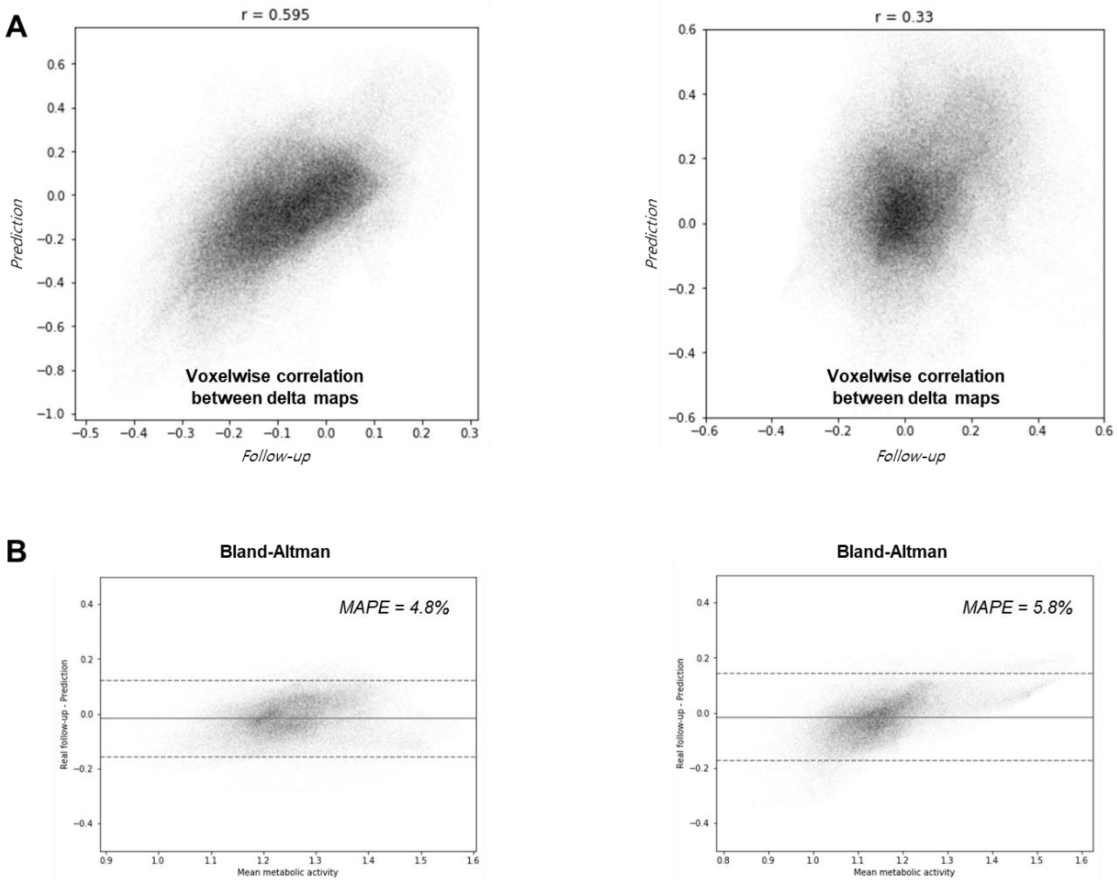

Supplementary Figure 2. Voxelwise correlation between predicted and real metabolic changes. (A) Voxelwise correlation between the delta maps of the two cases (represented in Fig. 1D) showed positive correlation (r = 0.60 and r = 0.33 for left and right cases, respectively). (B) Bland-Altman plots were drawn for voxelwise comparison between cortical metabolism of predicted and real follow-up data. Averaged errors of cortical metabolism of each voxel (MAPE: mean absolute percent error) were 4.8% (left) and 5.8% (right), respectively.

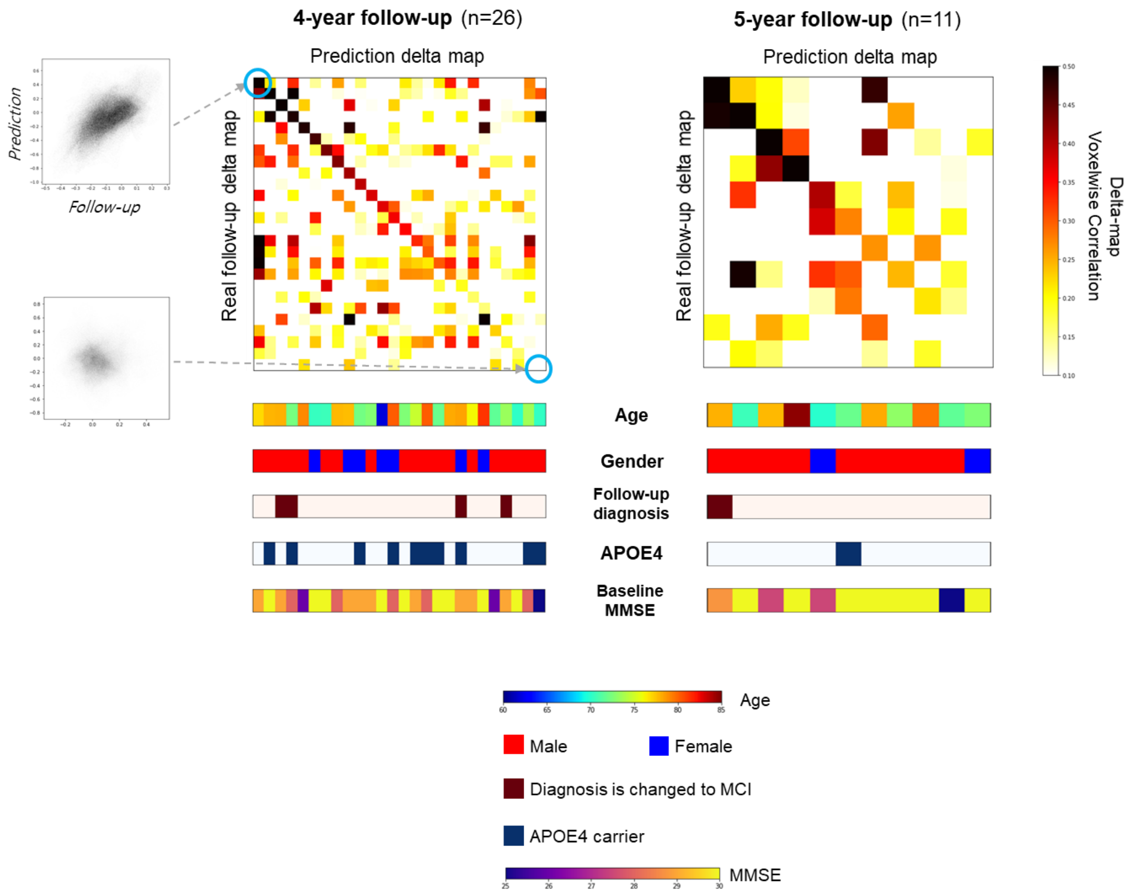

Supplementary Figure 3. Similarity between predicted and real metabolic changes for individual subjects. The voxelwise similarity between individual predicted and real metabolic change was assessed by correlation analysis. Individual delta maps obtained by follow-up data were correlated with those obtained by prediction and correlation matrix was generated. Diagonal components of the matrix represent correlation of delta maps of corresponding subjects. The correlation matrix was visualized for 4-year follow-up data (left) and 5-year follow-up data (right). Note that left upper components represent accurately predicted subjects while right lower components represent failed individual prediction. Clinical variables including age, gender, follow-up diagnosis, APOE4 status and MMSE score did not significantly affect the similarity between predicted and real metabolic changes.

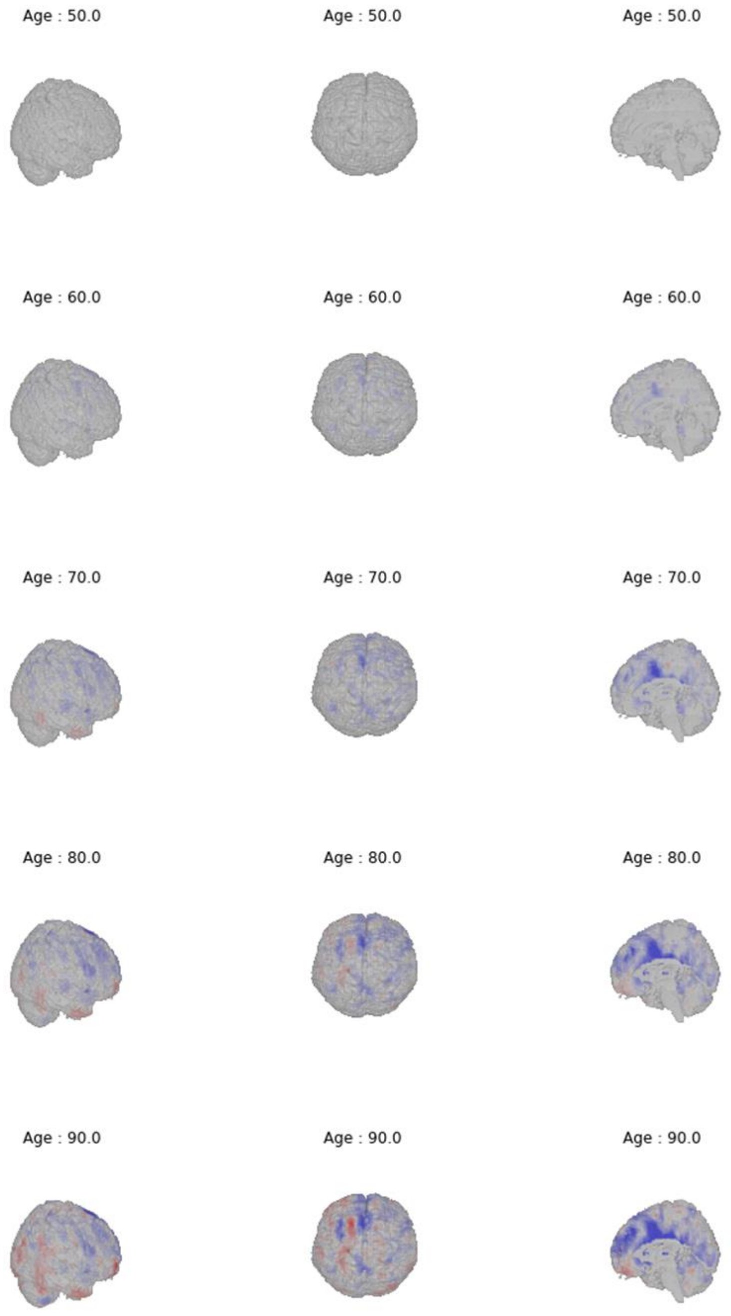

Supplementary Figure 4. Overall brain metabolism aging patterns. Surface visualization of the subtraction map compared with generated PET at 50-year-old was exhibited.

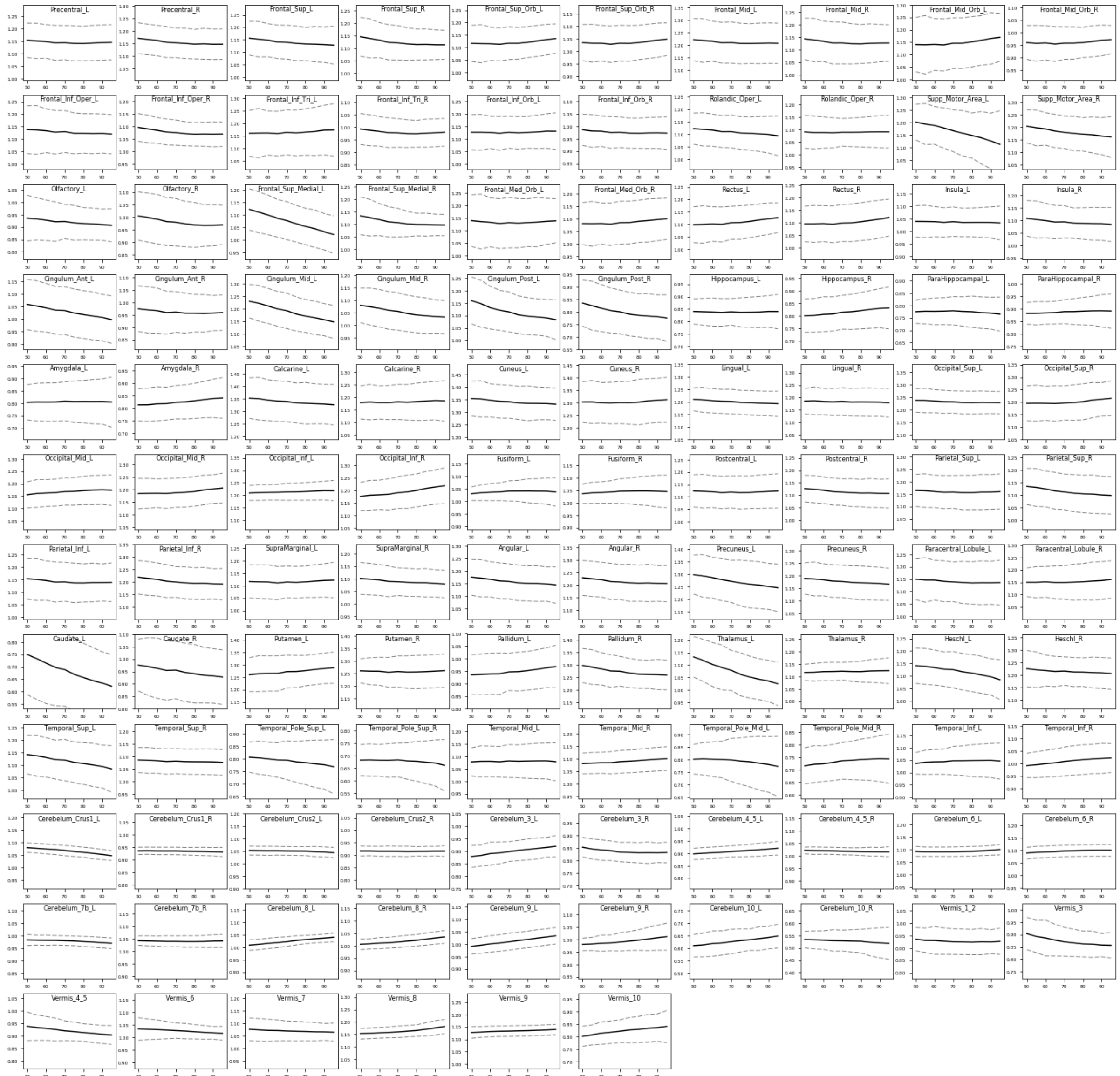

Supplementary Figure 5. Regional metabolic changes of all brain regions. Distribution of metabolic activity was estimated for all brain regions at different ages. Plots representing age-related metabolic changes were drawn for all brain regions. Dotted lines represent 95% confidence intervals.

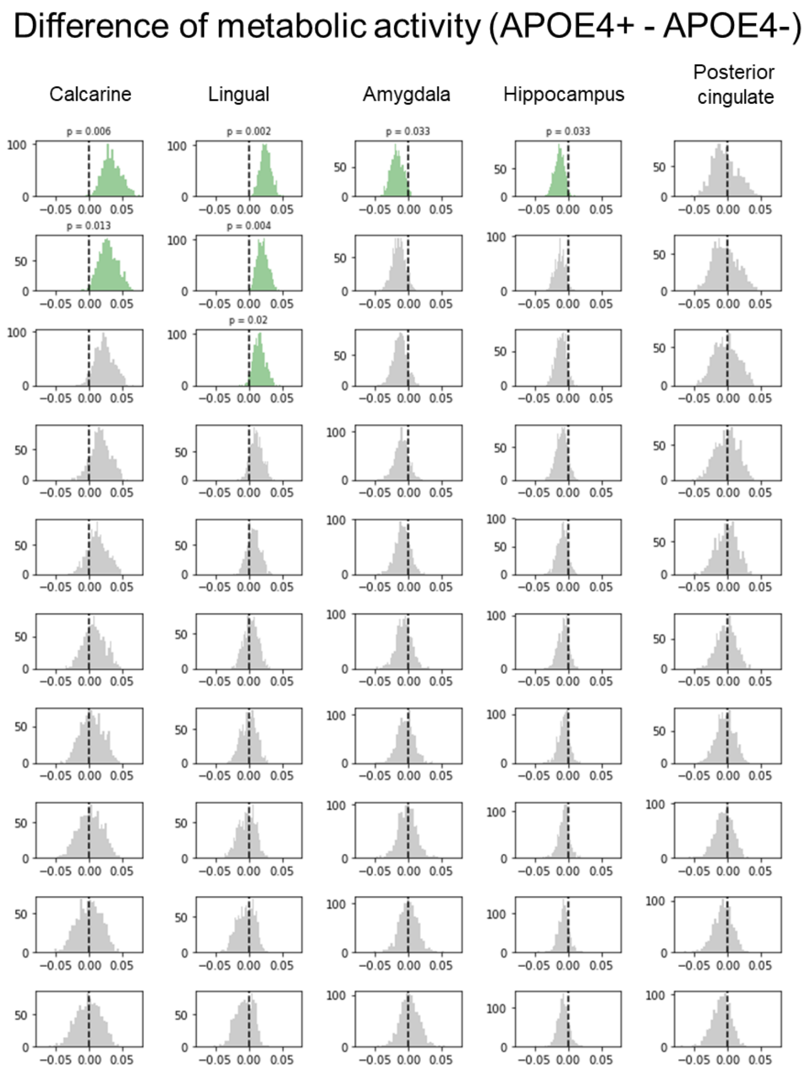

Supplementary Figure 6. Distribution of difference of regional metabolic activity between APOE4 carriers and noncarriers. Distribution of the regional metabolic difference was obtained by iteratively resampled PET generation. To obtain statistical significance, proportion of the difference less than or more than 0 was calculated. Histograms with green color represent significantly different regional metabolic activity.

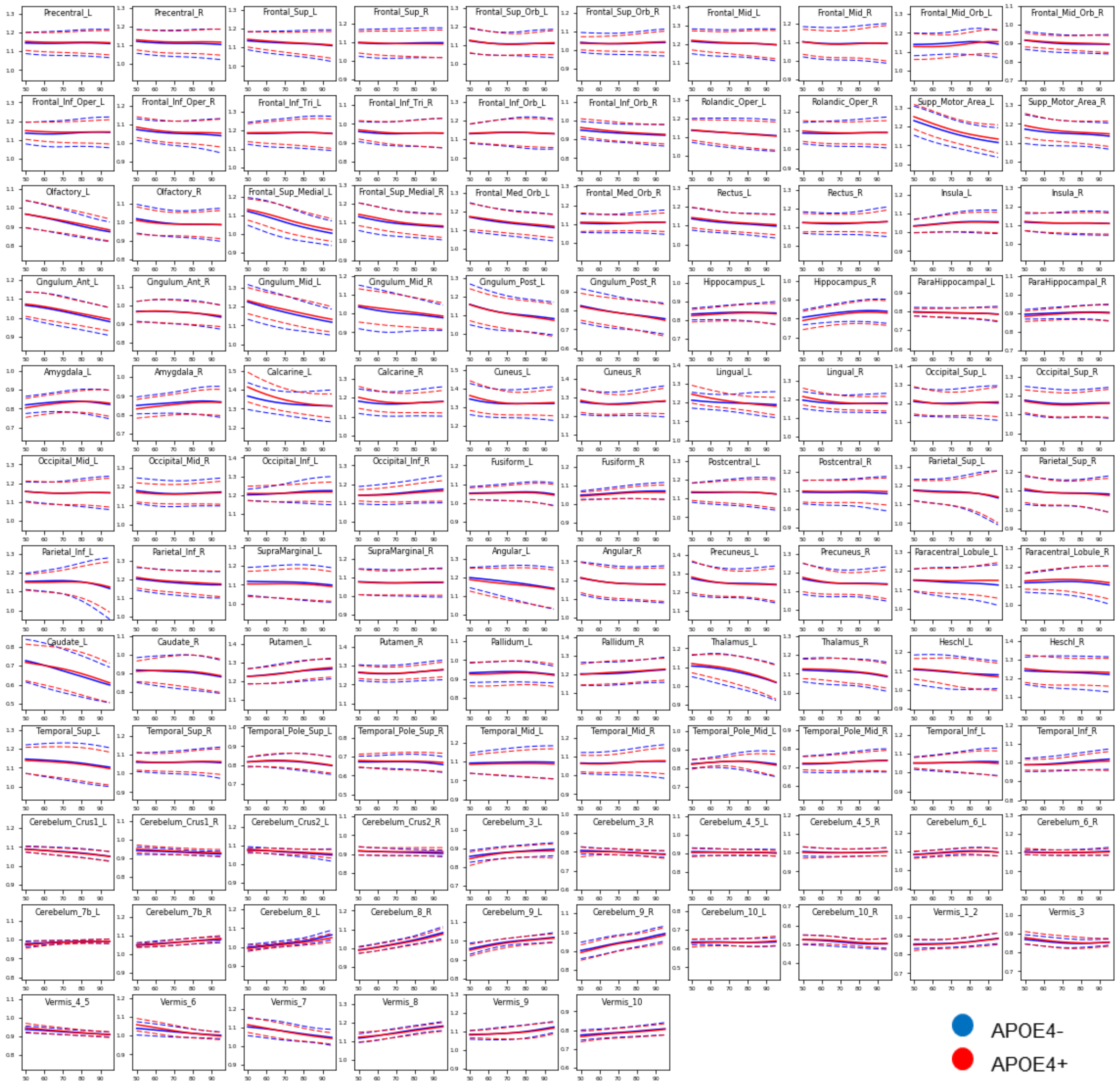

Supplementary Figure 7. Age-related metabolic changes according to APOE4 status. Regional metabolic activity changes of all brain regions were estimated by entering each APOE4 status into the generator. Red lines represent APOE4 carriers and blue lines represent APOE4 noncarriers. Dotted lines represent 95% confidence intervals computed by the resampled distribution.

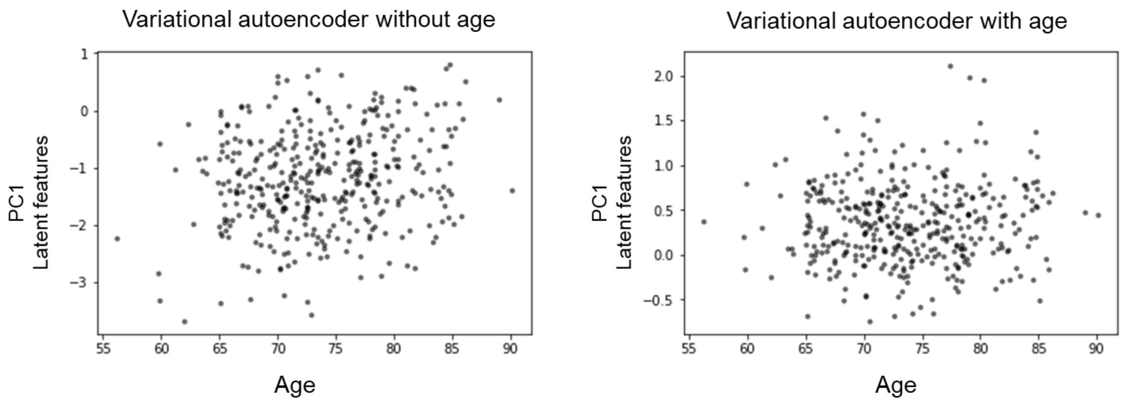

Supplementary Figure 8. Comparison of variational autoencoder without age and variational autoencoder with age. Encoded latent features were different between the model without age information and the model with age information. A principal component (PC1) was calculated from the latent features of each encoder. PC1 of the model without age was more correlated with age, which suggest the model without age extract image features related to aging (r = 0.20 for the model without age; r = -0.01 for the model with age).

2 Supplementary Movies

Supplementary Movie 1. Aging of representative brain metabolic topography

Supplementary Movie 2. Aging change of brain metabolism
